# Queue questions: Ethics of COVID‐19 vaccine prioritization

**DOI:** 10.1111/bioe.12858

**Published:** 2021-02-08

**Authors:** Alberto Giubilini, Julian Savulescu, Dominic Wilkinson

**Affiliations:** ^1^ Oxford Uehiro Centre for Practical Ethics University of Oxford Oxford United Kingdom of Great Britain and Northern Ireland; ^2^ Wellcome Centre for Ethics and Humanities University of Oxford Oxford UK; ^3^ Visiting Professorial Fellow in Biomedical Ethics Murdoch Childrens Research Institute Melbourne Australia; ^4^ Distinguished Visiting International Professorship in Law University of Melbourne Melbourne Australia

**Keywords:** COVID‐19, immunization, pandemic ethics, prioritization, vaccination ethics

## Abstract

The rapid development of vaccines against COVID‐19 represents a huge achievement, and offers hope of ending the global pandemic. At least three COVID‐19 vaccines have been approved or are about to be approved for distribution in many countries. However, with very limited initial availability, only a minority of the population will be able to receive vaccines this winter. Urgent decisions will have to be made about who should receive priority for access. Current policy in the UK appears to take the view that those who are most vulnerable to COVID‐19 should get the vaccine first. While this is intuitively attractive, we argue that there are other possible values and criteria that need to be considered. These include both intrinsic and instrumental values. The former are numbers of lives saved, years of life saved, quality of the lives saved, quality‐adjusted life‐years (QALYs), and possibly others including age. Instrumental values include protecting healthcare systems and other broader societal interests, which might require prioritizing key worker status and having dependants. The challenge from an ethical point of view is to strike the right balance among these values. It also depends on effectiveness of different vaccines on different population groups and on modelling around cost‐effectiveness of different strategies. It is a mistake to simply assume that prioritizing the most vulnerable is the best strategy. Although that could end up being the best approach, whether it is or not requires careful ethical and empirical analysis.

## 1 INTRODUCTION

At least three COVID‐19 vaccine candidates have now shown very promising preliminary results and either have been or are about to be approved for distribution in 2021 in most high‐income countries. The most urgent decision to be made now is about access prioritization. Actually, this will be an issue for as long as availability will not be enough to vaccinate a substantial proportion of the population. Initially, we will have vaccines for a very small proportion of the population.

Distribution decisions will have to be across two dimensions: global distribution among countries and national distribution among population groups. Here we will address the second question. For an individual country with an initial limited supply of vaccine, what criteria should be followed in deciding which population groups to prioritize, and why?

Prioritization decisions are first and foremost ethical decisions. They are not scientific decisions, although they are often presented as if they were. For instance, according to the UK Joint Committee on Vaccination and Immunisation (JCVI), which advises the UK Government on vaccine distribution, ‘mathematical modelling indicates that as long as an available vaccine is both safe and effective in older adults, they should be a high priority for vaccination’.^1^ This is ethical language disguised as scientific. Science can tell us how to achieve whatever we want to achieve with a vaccination policy. But what we want to achieve depends on which values we think matter the most.

The ethical nature of such decisions is most apparent when different possible goals can conflict with one another and we need to decide which goal to prioritize. For instance, we need to decide whether to use the first doses of the vaccine to save as many lives as possible from COVID‐19, to save certain kinds of lives rather than others (for example those that can be prolonged for the longest, or those that can be expected to be of good enough quality), to protect healthcare systems’ capacity, to promote the wider collective good, and so on. These are not necessarily the same goals and can conflict with one another.

It might be thought to be obvious that we ought to maximize the public health benefit of scarce medical resources such as vaccines. However, what counts as maximizing the public health benefit depends on what we take to be the relevant benefit and what portion of the population is the relevant ‘public’. Again, these are ethical questions, not scientific questions.

It is often taken for granted that the criterion for prioritizing access to COVID‐19 vaccines is vulnerability to COVID‐19: the most vulnerable should get the vaccine first. ‘Most vulnerable’ is often taken to mean those with the highest probability of dying if infected.

The JCVI suggests an adjusted age‐based ranking for prioritization (DHSC 2020). The ranking is aged‐based because age is the main risk factor and a good proxy for the presence of other underlying health conditions (e.g. diabetes, heart conditions, kidney failures) that represent risk factors. The ranking is adjusted to account for another very significant risk factor, which is residence in care homes, as well as the need to prioritize some essential workers, such as workers in care homes and healthcare workers. Thus, the prioritization order recommended by JCVI and that the UK Government is intentioned to follow is: ‘1. older adults’ resident in a care home and care home workers; 2. all those 80 years of age and over and health and social care workers; 3. all those 75 years of age and over’ and then younger age groups in descending order.

Prioritizing the most vulnerable in this way is an ethical decision. The JCVI criteria mean those with less expected time left to live—say, a 90‐year‐old man in a care home—are prioritized over those who are still relatively vulnerable to COVID‐19 but are likely to live longer —say, an otherwise healthy 70‐year‐old woman. But if we frame the options in this way, it may become apparent that this criterion cannot simply be taken for granted and is in need of some ethical justification.

Other countries are likely to take a similar approach. For instance, the German Federal Ministry of Health commissioned report^2^ recommends the following priority list:

1. Prevention of severe courses of COVID‐19 (hospitalisation) and deaths.
2. Protection of persons with an especially high work‐related risk of exposure to SARS‐CoV‐2 (occupational indication).
3. Prevention of transmission and protection in environments with a high proportion of vulnerable individuals and in those with a high outbreak potential.
4. Maintenance of essential state functions and public life.

From this, it concludes that ‘[a]s a result, priority should be given to those individuals at the highest risk of death and serious illness from a disease such as COVID‐19’.

Again, vulnerability to COVID‐19 is the main prioritization criterion. The Germans, however, are more explicit in justifying it also through the need to prevent hospitalizations and therefore burdens on the healthcare system, and not just through a duty to protect the vulnerable per se.

This widespread approach might or might not be ethically justified, but the values underlying this decision need to be made explicit and discussed, if we want prioritization policies to be ethical.

Some other policy or guideline documents do make the ethical nature of such decisions more explicit. For example, the WHO SAGE documents on allocation of COVID‐19 vaccines mention six principles that are relevant to vaccine distribution (human well‐being, equal respect, global equity, national equity, reciprocity, legitimacy).^3^ These ethical principles are helpful in determining priority. The WHO SAGE group describes three possible groups who should have the highest priority in case of very limited availability: (a) health workers and older adults in a situation of community transmission; (b) health workers and older adults in a situation in which there are sporadic cases or clusters of cases; (c) those more likely to spread the virus if infected in a situation in which there are no cases.

However, it is not clear how those principles generate these three priority groups. Moreover those principles are not exhaustive and are underspecified. How should we compare using limited vaccines to prevent community transmission vs. e.g. prevent outbreaks in specific settings such as work places or care homes? We would need other principles or values. And while those principles are certainly important, they need to be unpacked and further defined if they are to be applied. ‘Well‐being’ would translate into different prescriptions, depending on how it is defined, whose well‐being we are considering, duration of well‐being, and others factors we will consider in this paper.

In the same way, the US National Academy of Sciences, Engineering, and Medicine (NASEM)^4^ relies on the WHO SAGE principles and more prominently on the three ‘foundational’ ethical principles of maximum benefit, equal concern, and mitigation of health inequities. Again, these include important justice considerations that need to be taken into account, but these values also need to be further specified and defined. For example, the NASEM defines ‘maximum benefit’ in terms of ‘reduction of severe morbidity and mortality’ caused by COVID‐19, but as we shall argue in this paper, whether that counts as the maximization of the public health benefit of scarce COVID‐19 vaccines is in itself something that depends on ethical decisions that these documents leave either implicit or ambiguous.

Besides, regardless of whom we think we should protect from COVID‐19 in the first instance, we need to ask whether prioritizing this group in accessing the vaccine is the best way of achieving the goal. We will start by this latter question in the next section, before proceeding (Section 3) to discussing the various ethical considerations involved in setting the target for COVID‐19 vaccine distribution policies, distinguishing between the intrinsic (Subsection 3.1) and the instrumental (Subsection 3.2) value of different criteria.

## 2 WHOM TO PROTECT FIRST AND WHOM TO VACCINATE FIRST: TWO DIFFERENT QUESTIONS

Simply assuming that we should prioritize the most vulnerable seems to conflate the issues of prioritizing access to the vaccine and of prioritizing protection of a certain group. These are related, but are not necessarily the same issue. They might overlap in practice, but it is important to keep them conceptually distinct if we want to implement the most effective strategy possible for whatever goal we set.

Let us assume for the moment that protecting the most vulnerable should be the priority. Whether the most effective way of achieving this goal is to give the vulnerable priority access to the vaccine depends on the risk and effectiveness profile of the vaccine on different age groups, its ability to reduce transmission, and its availability.

For the flu vaccine, for example, we might best protect the vulnerable by actually prioritizing a different group. A strong case can be made for targeting children through vaccination in order to protect the elderly indirectly. This is based on the fact that the flu vaccine is more effective in the young than in the elderly (which increases the chances of having higher immunity at the collective level), it is very safe in the young (which arguably makes it ethically acceptable to administer to the young even if they have less to benefit from it), it effectively reduces transmission (which contributes to maximizing its expected utility by exploiting indirect protection), and is widely available, at least in high‐income countries (which increases the chances of successfully vaccinating enough people to create herd immunity and protect the elderly).^5^

The analogy with flu vaccination is only meant to emphasize the general point that the group we want to protect and the group we might need to target are not necessarily the same. Of course, there normally is no significant shortage of the flu vaccine at least in high‐income countries, so the parallel is not meant to suggest that COVID‐19 vaccination strategies should necessarily be the same as flu vaccination strategies.

We can consider two main scenarios with regard to the future COVID‐19 vaccines, and two possible variants of the second one.

*Scenario A*: *COVID‐19 vaccine is highly effective on the elderly*. If a COVID‐19 vaccine is likely to be very effective on the elderly (as currently appears to be the case with the ‘Pfizer vaccine’, on the basis of preliminary, non‐peer reviewed data^6^), then prioritizing vaccine distribution to the most vulnerable would likely be the most effective way of protecting them. Herd immunity could be built up as younger people mix and build natural immunity (with low mortality as they are at low risk) or with later availability of the vaccine.

*Scenario B*: *COVID‐19 is not very effective on the elderly but significantly more effective on the young*. If instead COVID‐19 vaccines are like the flu vaccines in being more effective in children or in some other young age groups, reduce community transmission, and are not very effective on the elderly, then giving it to the elderly first might paradoxically lead to more deaths in vulnerable groups. This strategy would be a waste of a scarce resource, at least in the initial phases of vaccine roll out where there will be relative shortage of vaccine. In such cases, an indirect protection strategy might be more effective.

At the moment, for example, although the Oxford/AstraZeneca vaccine is being rolled out in the UK (together with the Pfizer/BioNTech one) on older population groups, it is not clear whether the very high effectiveness of a certain dosage of it, which has so far been recorded on younger population groups, will be maintained in the elderly. More importantly, we do not know how long immunity will last and it is possible immunity will wane more quickly in the elderly (though at variable and unpredictable rates), leaving many of them vulnerable after a period of protection. Vaccinating younger age groups might then be a more effective and efficient way of ensuring persisting protection of the elderly.

Given certain assumptions about the COVID‐19 vaccine’s availability, effectiveness, and safety profile, it could be more effective to target the young in order to protect the elderly.^7^ This would be ethically justified even though the young would have less to benefit from the vaccine, given that COVID‐19 does not pose a significant threat to them. Even though the young would be used as a means to protect the old, a large collective benefit seems proportionate to the small individual vaccine related risks imposed on the young.^8^

There are two variations of this scenario.

*Scenario B1: Enough availability for herd immunity*. When we have enough availability to aim at herd immunity, the best strategy might be to administer the vaccine to the population group *more likely to respond to the vaccine*. If availability is high, protecting the most vulnerable could be achieved through building up general population herd immunity. While there still is uncertainty around what the threshold for herd immunity from COVID‐19 is, most estimates suggest it probably is around 60–70%.^9^ Vaccination policies should aim at that target when availability allows—which would also depend on what level of naturally conferred immunity already exists in the population and how long such immunity can be expected to last. Mathematical models suggest that a vaccine with an efficacy above 80% would prevent a resurgence of COVID‐19 cases if 70% of the population is vaccinated, in absence of non‐pharmacological measures (e.g. social distancing or face covering).^10^

*Scenario B2: Not enough availability to aim at herd immunity*. If, as currently seems more likely, initial availability will not be enough to aim at herd immunity, then the best way to protect the most vulnerable, given the same empirical assumptions, might be to prioritize their primary carers, both in their private homes and in care homes. We have to bear in mind that the most vulnerable are the very old in care homes, followed by the elderly not in care homes with certain pre‐existing health conditions. Vaccinating their carers would be practically equivalent to a form of ‘shielding’, with the additional benefits of safer interactions with primary carers.

In all these cases (type B), we would need to be confident that the vaccine prevents transmission in those vaccinated, and is sufficiently safe for the targeted group to make any small risks worth the benefits of the vaccine.

## 3 INTRINSIC AND INSTRUMENTAL VALUES

There are two reasons to protect the most vulnerable first. First, because we want to save as many lives as possible from COVID‐19. Second, because we want to reduce the burden on the public health system posed by those who, if infected, would require hospitalization and life‐saving treatments.

These are two different reasons. As we saw above, the UK approach is (at least explicitly) mostly based on the former while the German approach also puts some emphasis on the latter. The first reason is based on some intrinsic value attributed to human life and on some moral imperative to save lives from imminent threats. The second is based on the instrumental value of saving lives, as a means to saving scarce healthcare resources, and ultimately saving more lives overall, both from COVID‐19 and non‐COVID‐19 related illnesses (even if the threat from the latter is not imminent).

In practice, these might well overlap, at least given certain assumptions. However, one reason why they do not necessarily overlap is that not every patient who might become seriously unwell or die from COVID‐19 would necessarily receive treatment in hospital or in an intensive care unit. For example, some patients who are in residential care may have advance care plans indicating that they would not wish to be treated in hospital or ventilated if they developed pneumonia. They may nevertheless wish not to die from COVID‐19 in the short term. If we wish to save the most lives, such patients should potentially be prioritized for the vaccine. However, if we wish to reduce burden on the healthcare system, they would not receive priority. If they get infected with COVID‐19, they would not be using scarce intensive care resources, and therefore a COVID‐19 vaccine allocation policy aiming at preserving availability of intensive care units would not prioritize these groups.

If we are focused on the intrinsic value of the goal of vaccine distribution, there is a further question about what that should be.

### 3.1 Intrinsic values

#### 3.1.1 Intrinsic value 1: Saving as many lives as possible from COVID‐19?

If the ultimate goal is to save as many lives as possible, then it means that what we value most is saving lives. This is because we think life has such intrinsic value that we should try to save as many lives as possible.

According to current models,

[w]hen structuring by age alone, the most efficacious reduction was found through an oldest first approach – despite not being the most crucial group in terms of transmission, the considerably heightened vulnerability amongst the elderly means that priority should be given to protecting them directly.^11^

According to some, this largely aged based prioritization model can plausibly be taken to be the rationale behind the UK prioritization criteria.^12^ However, reduction of life loss was maximized by this strategy only on the assumption that the vaccine has high efficacy in protecting the vaccinated, as well as on the alternative assumption that the vaccine is less effective at protecting the vaccinated but effective at preventing transmission. Under such assumptions, the best strategy if the priority is to save lives is to prioritize those over 80 years of age, followed by healthcare workers, and then younger age groups in descending order.^13^

It might seem obvious that we have a duty to save as many lives as possible. But it is worth noting that this is not the ethical principle that normally guides allocation of scarce healthcare resources and public health decisions more generally. As we shall see in more detail below, the NHS in England allocates life‐saving treatments on the basis of considerations of cost per quality‐adjusted life‐year (QALY). This means that the expected quality and length of the life that could be saved are a relevant consideration in determining whom to prioritize in access to life saving treatments. The criterion adopted is not simply that of saving as many lives as possible.

#### 3.1.2 Intrinsic value 2: Saving years of life?

Under many ethical views, saving the life of a person who then goes on to die the following day is ethically different from saving the life of person who then goes on to live another 40 good years.

When it comes to choices of treatment for ourselves, we would not be agnostic about choosing between a treatment that might prolong our life for a very short time and one that would give us many years of life. Thus, we might think that what matters is not life itself and therefore how many lives we save from COVID‐19, but how much time we are buying to each person who is saved. The most vulnerable to COVID‐19 are the elderly. The older you are, the higher the risk of dying from COVID‐19, other things being equal, which explains why the JCVI ranking is predominantly aged‐based.

However, the older you are, the less time you are likely to gain by being saved from COVID‐19.

The general public appears to support consideration of this factor in provision of scarce life‐saving treatment in the pandemic. Presented with a series of triage dilemmas, in the face of limited intensive care resources, members of the UK general public overwhelmingly chose to prioritize to treat patients with a longer life expectancy.^14^

If you are an over 85 years old man, your risk of dying from COVID‐19 is relatively high, somewhere between 10% and 27%, even without underlying health conditions. If the vaccine saves you from COVID‐19 (either directly or indirectly), you could expect to live another 6 years. If you are a 70‐year‐old woman, your risk of dying from COVID‐19 is lower, somewhere between 3% and 11%, again absent any other risk factor. But if the vaccine saves you from COVID‐19, you can expect to live another 18 years.^15^

If we want to prioritize the vulnerable, and if direct immunization is the best way to protect the vulnerable, we should prioritize the over 85‐year‐old man.

But if we want to prioritize those who have more to benefit in terms of life years saved, we should potentially prioritize the 70‐year‐old woman (assuming the chance of an 85 year old dying is less than three times that of a 70 year old).

The picture becomes more complicated when we consider other aspects of the current scenario. The most vulnerable to COVID‐19 are the elderly in care homes. In a country like the UK, the average length of people’s stay in a care home before they die is slightly more than 2 years (801 days), though with significant variations (e.g. more than a quarter live for longer than 3 years).^16^

Again, if we want to simply prioritize the vulnerable (through direct immunization) then this factor should be disregarded. But if we think it matters for how long a person can be expected to live if they do not die from COVID‐19, then a factor like residency in care homes would make a significant difference to initial vaccine allocation, though in the opposite direction to the one guiding current prioritization lists in the UK.

#### 3.1.3 Intrinsic value 3: Quality of life?

We might think that what matters is not only the number of lives we save and for how long we save them, but also the quality of the lives we save. It is one thing to save from COVID‐19 someone whose life, quite regardless of its length, is going to be extremely limited in quality, and it is quite another thing to save from COVID‐19 someone whose life is going to be fulfilling and valuable. In the extreme, it is a low priority to spend limited resources on somebody who is permanently unconscious, compared to a person who is in full possession of their mental faculties. When resource availability is not an issue, we might have ethical reasons to save all these types of lives (except, where the individual would not wish their life to be saved). However, when we have to make that choice because we cannot save everyone, some would judge this factor as more relevant.

Again, whether or not we take it into account is an ethical choice. It depends on whether we confer some intrinsic value to human life or whether we think the ethical value of human life depends on its quality, and on what criteria we use to assess the quality of certain lives.

For example, in the UK 70% of residents in nursing homes have some form of dementia,^17^ which is very debilitating especially at the late stage. Although various factors affect quality of life (QoL) of people with dementia and many patients do not experience significant deterioration of QoL,^18^ dementia is often accompanied by severe anxiety and depression and ultimately deterioration of QoL.^19^ In the extreme, dementia results in unconsciousness where there is arguably zero quality of life and the patient no longer has interests.^20^

In the UK pandemic triage survey mentioned above, a large majority of the general public elected to prioritize intensive care to a patient with no disability rather than a patient with a profound learning disability.^21^

Any decision to preferentially allocate a vaccine based on factors affecting quality of life (particularly diseases and disabilities) is likely to be perceived as controversial and even discriminatory.^22^ However, whether it is unfair or otherwise unethical is a different matter. It depends on whether we want to use an egalitarian principle (i.e. everyone, or everyone within the same age group, is given the same chance to access the vaccine, e.g. through a lottery) or some other principle, such as a utilitarian principle (i.e. we should maximize the overall benefit of the vaccine, and the notion of ‘benefit’ requires to take into account the quality of the lives we save) or a contractualist principle based on what we would choose ‘from behind a veil of ignorance’, i.e. not taking into account our personal circumstances and therefore not knowing our risk of dying or suffering serious consequences from COVID‐19. Again, this is an ethical decision.

#### 3.1.4 Intrinsic value 4: Quality‐adjusted life‐years (QALYs)?

The two previous values—length and quality of life—are often combined in the concept of expected quality‐adjusted life‐years (QALYs), a criterion that discounts the value of additional years of life on the basis of deterioration in their quality. A QALY is very simply calculated by multiplying years of life by their quality on a scale from 0 to 1. This is often considered a useful criterion in decisions about allocation of scarce healthcare resources, and in public health policy more generally. For example, the current policy in the UK is that treatments that cost more than £30,000 per QALY saved are unlikely to be provided by the NHS.^23^ While this figure might not be used directly in deciding how we should regulate priority access to COVID‐19 vaccines, it does suggest that QALYs are indeed already considered an ethically relevant criterion for allocation of scarce health resources.

There have been models of the cost per QALY of different prioritization strategies for COVID‐19 vaccination. One modelling paper from the US predicts that, overall, targeting groups at higher risk of hospitalization and death from COVID‐19 would make vaccination more cost‐effective, other things being equal. Vaccination in their analysis became less cost‐effective as the risk of hospitalization/death decreased. The vaccine still fell within cost‐effectiveness thresholds if targeted at patients age 50–64, but was associated with a high cost/QALY for younger patients (in the absence of additional risk factors).^24^ According to the aforementioned model that supported the UK prioritization strategy, prioritizing those over 80 is the best strategy not only to maximize the number of lives saved from COVID‐19, but also to maximize QALYs saved, on the assumption that the vaccine is highly effective at both protecting the vaccinated from COVID‐19 and stopping transmission.^25^ While this model does not mention cost‐effectiveness explicitly, it is plausible to assume that the same considerations apply once costs are considered.^26^

Which age group to target would depend on what weight (if any) we want to give to QALYs as a criterion for effectiveness of a vaccination policy, and what weight we want to give to cost‐effectiveness in terms of QALYs compared to the other values at stake. For example, we might want to make the vaccine slightly less cost‐effective in terms of QALYs and give some weight to one of the alternative values here discussed, including some of the instrumental values below.

Including QALYs in the ethical assessment of prioritization in access to COVID‐19 vaccines would have a significant impact on the currently endorsed prioritization rankings. To use the same example as above, dementia reduces both length and quality of life and would therefore significantly affect an individual’s expected QALYs. That could mean, for example, that patients with severe dementia should have a lower priority.

Again, whether or not QALYs should be considered as one of the prioritization criteria, and what weight they should be given, is an ethical decision. If we want this policy to be consistent with other policies around allocation of scarce healthcare resources, QALYs should at least be considered.

Of course, we might think that, as has been pointed out, ‘the unprecedented scale of the pandemic invalidates the usual metrics in this approach’^27^ and their ethical value. But whether and to what extent we should rely on the usual metrics, also in light of our possible diminished confidence in their accuracy, is in itself an ethical decision that would need to be made.

#### 3.1.5 Other intrinsic values

Inclusion of length or quality of life would be consistent with allocation of other medical treatments in many publicly funded healthcare systems. However, there may be other values that could be included in allocation. For example, in intensive care triage, some ethicists have advocated giving priority to patients who have not yet had the chance to live through different phases of the life cycle.^28^ Presented with hypothetical triage dilemmas, members of the UK public gave priority to younger over older patients, even if the patients had identical life expectancy.^29^ In that study, a further factor considered relevant to prioritization by the public was whether patients had dependants (see section on ‘Instrumental values’ below).

If those values were incorporated into vaccine allocation, that might mean that the JCVI prioritization list should be revised to give greater priority to younger patients. This could include particularly those younger patients who are also clinically vulnerable (who might have a similar mortality risk to the elderly).

### 3.2 Instrumental values

#### 3.2.1 Instrumental value 1: Protecting the healthcare system?

If the ultimate goal is to avoid overwhelming public healthcare systems, and protecting the most vulnerable is instrumental to achieving this goal, then aiming at protecting the most vulnerable may be the right approach—whether we do it by direct or indirect protection strategies, as we said above.

The aforementioned survey of attitudes to intensive care triage^30^ indicated that the public would support giving priority to front‐line healthcare workers. Again, this seems to be justified by some instrumental value of protecting a specific group. In this case, prioritizing healthcare workers might be justified in several complementary ways—for example, to maintain an essential service, to reduce transmission to vulnerable patients, because such workers are potentially at elevated risk of more serious illness, and as a form of compensation for the risks/burdens taken on by healthcare workers.

#### 3.2.2 Instrumental value 2: Broader societal interests

We have seen that one possible reason for targeting the most vulnerable as the priority group is to preserve public health systems’ ability to provide healthcare to the largest number of people possible, without being overburdened by COVID‐19 patients. This is because healthcare provision is considered an essential service for the collective.

However, there are other essential services a society needs and that it is in the collective significant interest to protect from the burdens imposed by COVID‐19.

Certain criteria were adopted to identify ‘key workers’ who would be exempted from lockdown restrictions because of the essential nature of their services. The same criteria could be used to guide prioritization in vaccination policies for exactly the same reason, ideally in proportion to the level of risk that their activities expose them to (both in terms of being infected and infecting others).

According to the UK Government criteria, these include not only workers in health and social care (some of whom are already included in the prioritization criteria mentioned above), but also those working in certain areas of education and childcare, utilities and communication, food and necessary good, transport, key public services, public safety and national security, and in national and local governments.^31^

The risk profiles across and within these groups might be different. However, the same consideration of collective interest that grounds the choice to prioritize healthcare workers would also require the prioritization of COVID‐19 vaccination at least for some of these groups, in proportion to the extent to which they would protect significant collective interests.

According to the same criterion, a group with higher priority might need to be those with dependants, e.g. parents of young children, and among these those with dependants in high risk groups for COVID‐19. Again, in such cases prioritizing certain groups would be instrumental to protecting other groups’ interests (e.g. young children’s interest in having adequate care and support or vulnerable dependants’ interests in minimizing their exposure to coronavirus).

France will include among the high priority groups, after health workers, professions such as ‘shop workers, school staff, transport staff and hospitality workers, as well as those working in confined spaces such as abattoir staff, taxi drivers, migrant workers and construction teams’.^32^ The criterion adopted here is the risk of spreading the virus or being infected. This might be a sensible thing to do, but it is worth emphasizing that, also in this case, targeting these groups is instrumental to pursuing some broader societal interest (presumably, reducing the risk of infection among vulnerable groups and keeping these services safe and operative).

Prioritizing vaccine access on the basis of individuals’ ‘societal value’ might sound ethically suspicious to many people and applying the criterion consistently might lead to some counterintuitive measures. However, that the societal values of individuals should be given moral weight in decisions about ‘whom to save when you cannot save them all’ has quite large public support.^33^

## 4 CONCLUSION

We ought to maximize the public health benefit of the initially limited availability of the future COVID‐19 vaccines. It is often assumed—including in policy making—that this is achieved by protecting the most vulnerable first and therefore by giving them priority access to the COVID‐19 vaccines. Given what we know about the COVID‐19 fatality rate, this means prioritizing people based on age and residency in care homes.

Here we have shown that this criterion, even if often framed in technical scientific terms (especially in the JCVI document mentioned above), is actually an ethical criterion that presupposes certain choices about value. But the weight placed on such values can be questioned.

Whether we prioritize the young or the elderly to receive the vaccine will depend on facts about the effectiveness of the vaccine in saving life and reducing transmission at different ages, but also on what we want to achieve.

What counts as maximization of public health benefits depends on who counts as the relevant ‘public’ and what counts as a benefit.

The relevant public could be the most vulnerable, or those with longer life expectancy and significantly high risk from COVID‐19, or those who can expect to have a good enough quality of life, or those who depend for various reasons on other people’s services, or the collective more broadly.

The relevant benefit could be saving as many as possible from COVID‐19, prolonging lives to a significant extent and/or prolonging lives of a good enough quality, protecting a public healthcare system, protecting a society’s essential services.

Some of the aforementioned factors enjoy the widespread support of lay people in UK and around the world.

Any choice we make requires making ethical decisions. What counts as a public health benefit is itself an ethical decision. Saving lives is only one ethical value among many that ought to be considered.

## CONFLICT OF INTEREST

The authors declare no conflict of interest.

## Footnotes

^1^

Department of Health and Social Care (DHSC) (2020). *JCVI: Updated interim advice on priority groups for COVID‐19 vaccination*. https://www.gov.uk/government/publications/priority‐groups‐for‐coronavirus‐covid‐19‐vaccination‐advice‐from‐the‐jcvi‐25‐september‐2020/jcvi‐updated‐interim‐advice‐on‐priority‐groups‐for‐covid‐19‐vaccination#references

^2^

Standing Committee on Vaccination (STIKO), The German Ethics Council (GEC) and The National Academy of Sciences Leopoldina (NASL) (2020). *How should access to a COVID‐19 vaccine be regulated?* https://www.leopoldina.org/uploads/tx_leopublication/2020_position‐paper‐vaccine‐prioritisation_ENG_final_01.pdf

^3^

WHO SAGE. (2020a). *Roadmap for prioritizing uses of COVID‐19 vaccines in the context of limited supply*. https://www.who.int/publications/m/item/who‐sage‐roadmap‐for‐prioritizing‐uses‐of‐covid‐19‐vaccines‐in‐the‐context‐of‐limited‐supply; WHO SAGE. (2020b). *WHO SAGE value framework for the allocation and prioritization of COVID‐19 vaccination*. https://apps.who.int/iris/bitstream/handle/10665/334299/WHO‐2019‐nCoV‐SAGE_Framework‐Allocation_and_prioritization‐2020.1‐eng.pdf?ua=1

^4^

NASEM. (2020). *Framework for equitable allocation of COVID‐19 vaccine*. https://www.nap.edu/catalog/25917/framework‐for‐equitable‐allocation‐of‐covid‐19‐vaccine 33026758

^5^

Bambery, B., Douglas, T., Selgelid, M. J., Maslen, H., Giubilini, A., Pollard, A. J., & Savulescu, J. (2018). Influenza vaccination strategies should target children. *Public Health Ethics, 11*(2) 221–234.10.1093/phe/phx021PMC609344030135702

^6^

See e.g. BBC. (Nov 18, 2020). Covid vaccine: Pfizer says '94% effective in over‐65s'. https://www.bbc.co.uk/news/health‐54986208

^7^

Bambery et al., op. cit. note 5.

^8^

Giubilini, A., Savulescu, J., & Wilkinson, D. (2020). COVID‐19 vaccine: Vaccinate the young to protect the old? *Journal of Law and the Biosciences, 7*(1), lsaa050.10.1093/jlb/lsaa050PMC733775932959006

^9^

Anschwanden, C. (2020). The false promise of herd immunity for COVID 19. *Nature, 587*, 26–28; Gomes, M. G. M., Corder, R. M., King, J. G., Langwig, K. E., Souto‐Maior, C., Carneiro, J., Gonçalves, G., Penha‐Gonçalves, C., Ferreira, M. U., & Aguas, R. (2020). Individual variation in susceptibility or exposure to SARS‐CoV‐2 lowers the herd immunity threshold. medRxiv. 10.1101/2020.04.27.20081893 PMC885566135189135

^10^

Moore, S., Hill, E. M., Dyson, L., Tildesley, M., & Keeling, M. J. (2020). Modelling optimal vaccination strategy for SARS‐CoV‐2 in the UK. medRxiv. 10.1101/2020.09.22.20194183 PMC810195833956791

^11^

Ibid.

^12^

Raftery, J. (2020, November 17). Who should be first in line for a covid‐19 vaccine? Assessing effectiveness and cost effectiveness. *British Medical Journal Opinion*. https://blogs.bmj.com/bmj/2020/11/17/james‐raftery‐who‐should‐be‐first‐in‐line‐for‐a‐covid‐19‐vaccine‐assessing‐effectiveness‐and‐cost‐effectiveness/

^13^

Moore et al., op. cit. note 10.

^14^

Wilkinson, D., Zohny, H., Kappes, A., Sinnott‐Armstrong, W., & Savulescu, J. (2020). Which factors should be included in triage? An online survey of the attitudes of the UK general public to pandemic triage dilemmas. *BMJ Open*, *10*(12), e045593. 10.1136/bmjopen-2020-045593 PMC772508733293401

^15^

See the Office for National Statistic’s Life Expectancy Calculator at https://www.ons.gov.uk/peoplepopulationandcommunity/healthandsocialcare/healthandlifeexpectancies/articles/lifeexpectancycalculator/2019‐06‐079 and the QxMD COVID‐19 Prognostic Tool Calculator at https://qxmd.com/calculate/calculator_731/covid‐19‐prognostic‐tool

^16^

Forder, J., & Fernandez, J.‐L. (2011). *Length of stay in care homes*. Report commissioned by Bupa Care Services, PSSRU Discussion Paper 2769, Canterbury: PSSRU.

^17^

Prince, M., Knapp, M., Guerchet, M., McCrone, P., Prina, M., Comas‐Herrera, A., Wittenberg, R., Adelaja, B., Hu, B., King, D., Rehill, A., & Salimkumar, D. (2014). *Dementia UK: Update*. Alzheimer’s Society. https://www.alzheimers.org.uk/sites/default/files/migrate/downloads/dementia_uk_update.pdf

^18^

Mjørud, M., Røsvik, J., Rokstad, A. M., Kirkevold, M., & Engedal, K. (2014). Variables associated with change in quality of life among persons with dementia in nursing homes: A 10 months follow‐up study. *PLoS ONE*, *9*(12), e115248. 10.1371/journal.pone.0115248; Oudman, E., & Veurink, B. (2014). Quality of life in nursing home residents with advanced dementia: A 2‐year follow‐up. *Psychogeriatrics: The Official Journal of the Japanese Psychogeriatric Society, 14*(4), 235–240. 10.1371/journal.pone.0115248

^19^

Hoe, J., Hancock, G., Livingston, G., Woods, B., Challis, D., & Orrell, M. (2009). Changes in the quality of life of people with dementia living in care homes. *Alzheimer Disease and Associated Disorders, 23*(3), 285–290. 10.1097/WAD.0b013e318194fc1e PMC275965619812472

^20^

Airedale N.H.S. Trust v Bland. (1993). A.C. 789 House of Lords. https://www.e‐lawresources.co.uk/Airedale‐N‐H‐S‐‐Trust‐v‐Bland.php

^21^

Wilkinson, D., Petrou, S., & Savulescu, J. (2018). Expensive care? Resource‐based thresholds for potentially inappropriate treatment in intensive care. *Monash Bioethics Review, 35*, 2–23.10.1007/s40592-017-0075-5PMC609686929349753

^22^

Savulescu, J., Cameron, D., & Wilkinson, D. (2020). Equality or utility? Ethics and law of rationing ventilators. *British Journal of Anaesthesia, 125*(1), 10–15.10.1016/j.bja.2020.04.011PMC716754332381261

^23^

Kirkdale, R., Krell, J., O'Hanlon Brown, C., Tuthill, M., & Waxman, J. (2010). The cost of a QALY. *QJM: An International Journal of Medicine, 103*(9), 715–720.10.1093/qjmed/hcq08120519275

^24^

Kohli, M., Maschio, M., Becker, D., & Weinstein, M. C. (2021). The potential public health and economic value of a hypothetical COVID‐19 vaccine in the United States: Use of cost‐effectiveness modeling to inform vaccination prioritization. *Vaccine*. 10.1016/j.vaccine.2020.12.078 PMC783265333483216

^25^

Moore et al., op. cit. note 10.

^26^

Raftery, op. cit. note 12.

^27^

Moore et al., op. cit. note 10.

^28^

Emanuel, E. J., Persad, G., Upshur, R., Thome, B., Parker, M., Glickman, A., Zhang, C., Boyle, C., Smith, M., & Phillips, J. P. (2020). Fair allocation of scarce medical resources in the time of covid‐19. *New England Journal of Medicine*, *382*(21), 2049–2055. 10.1056/nejmsb2005114 32202722

^29^

Wilkinson et al., op. cit. note 14.

^30^

Ibid.

^31^

ONS (Office for National Statistics). (2020). *Coronavirus and key workers in the UK*. https://www.ons.gov.uk/employmentandlabourmarket/peopleinwork/earningsandworkinghours/articles/coronavirusandkeyworkersintheuk/2020‐05‐15

^32^

Roope, L., Clarke, P., & Duch, R. (2020, November 16). Who should get the coronavirus vaccine first? *The Conversation*. https://theconversation.com/who‐should‐get‐the‐coronavirus‐vaccine‐first‐france‐and‐the‐uk‐have‐different‐answers‐149875

^33^

Awad, E., Dsouza, S., Kim, R., Schulz, J., Henrich, J., Shariff, A., Bonnefon, J.‐F., & Rahwan, I. (2018). The moral machine experiment. *Nature*, *563*(7729), 59–64. 10.1038/s41586-018-0637-6 30356211

